# The Taboo Against Explicit Causal Inference in Nonexperimental Psychology

**DOI:** 10.1177/1745691620921521

**Published:** 2020-07-29

**Authors:** Michael P. Grosz, Julia M. Rohrer, Felix Thoemmes

**Affiliations:** 1 Department of Psychology, University of Münster; 2 International Max Planck Research School on the Life Course, Max Planck Institute for Human Development; 3 Department of Psychology, University of Leipzig; 4 Department of Human Development, Cornell University

**Keywords:** causal inference, observational studies, nonexperimental, instrumental-variable estimation

## Abstract

Causal inference is a central goal of research. However, most psychologists refrain from explicitly addressing causal research questions and avoid drawing causal inference on the basis of nonexperimental evidence. We argue that this taboo against causal inference in nonexperimental psychology impairs study design and data analysis, holds back cumulative research, leads to a disconnect between original findings and how they are interpreted in subsequent work, and limits the relevance of nonexperimental psychology for policymaking. At the same time, the taboo does not prevent researchers from interpreting findings as causal effects—the inference is simply made implicitly, and assumptions remain unarticulated. Thus, we recommend that nonexperimental psychologists begin to talk openly about causal assumptions and causal effects. Only then can researchers take advantage of recent methodological advances in causal reasoning and analysis and develop a solid understanding of the underlying causal mechanisms that can inform future research, theory, and policymakers.

Correlation does not imply causation. This truism justifiably reminds researchers that they should not carelessly draw causal conclusions on the basis of nonexperimental evidence. However, instead of motivating psychologists to exercise due diligence and face the challenges of causal inference, it seems to have resulted in a widespread taboo against explicit causal inference in nonexperimental settings. This taboo has resulted in a dilemma in some fields of psychology. On the one hand, causal relationships are of central interest; on the other hand, they are “forbidden” when experiments are unfeasible or unethical. As a result, one might expect nonexperimental researchers to limit themselves to descriptive or predictive research questions. But nonexperimental researchers do not actually avoid asking causal research questions or drawing causal conclusions; it simply happens implicitly, opaquely, and without an articulation of the underlying assumptions.

Here, we argue that the taboo against explicit causal inference hinders nonexperimental psychology (for similar arguments, see Antonakis, Bendahan, Jacquart, & Lalive, 2010, and Hernán, 2018a). It impairs study design and data analysis. It slows the pace at which our understanding of underlying causal mechanisms grows. It leads to a disconnect between original studies and how they are integrated into subsequent work, and it limits the usefulness of nonexperimental psychology for policymaking. We elaborate on each of these points and suggest how nonexperimental psychologists can integrate causality into their work in a more productive manner.

## Manifestation of the Taboo

To illustrate the taboo against causal inference, we annotated text passages from four nonexperimental articles in Table 1. In all four articles, causal inference seems to be intended but are not expressed in explicit and straightforward ways, which we interpret as signs of the taboo. The absence of explicit causal language thus obscured the research goals of the studies. The researchers ended up sending mixed messages. Some parts of the articles read as if the entire endeavor were noncausal; yet other parts make sense only in the context of trying to answer a causal research question. For example, two of the four examples (Table 1, Examples 1 and 3) included control variables to rule out confounding effects and estimate the unique effect of the independent variable. Although there might be instances in which a third variable adjustment is useful for descriptive purposes, the adjustment in these two studies suggests that their goal was not merely to describe or to predict. If description were the ultimate goal, then an unadjusted estimate could have been reported, potentially with some insightful graphical display. At the same time, prediction did not seem to be the goal either, given that attention was paid to the coefficients of particular predictors rather than to the overall accuracy and cross-validation of the prediction. If prediction were the ultimate goal, then techniques from the field of machine learning would have been more appropriate (Yarkoni & Westfall, 2017). Hence, it is plausible to assume that the two examples that used control variables and even the two examples without control variables (Table 1, Examples 2 and 4) intended to identify causal links, even though the results of the studies were interpreted with vague causal language (e.g., “predict,” “effect”) rather than explicit causal language (e.g., “causes,” “causal effect”; for a similar argument, see Rutter, 2007). We think that description and prediction are worthwhile research goals. Yet we believe they should be pursued for their own sake rather than serving as a cover for implicit causal questions and conclusions.

**Table 1.** Manifestations of the Taboo Against Explicit Causal Inference in Four Nonexperimental Articles

Place	Content of text passage	Annotation
Example 1 (Moffitt et al., 2011)		
Title	“A gradient of childhood self-control predicts health, wealth, and public safety.” (p. 2693)	The title indicates that the research question is not causal but predictive in nature (i.e., the study intends to predict health, wealth, and public safety). That said, when prediction is the goal, then the focus is usually not on individual predictors (e.g., self-control).
Abstract	“Policy-makers are considering large-scale programs aimed at self-control to improve citizens’ health and wealth and reduce crime. Experimental and economic studies suggest such programs could reap benefits. Yet, is self-control important for the health, wealth, and public safety of the population? Following a cohort of 1,000 children from birth to the age of 32 y, we show that childhood self-control predicts physical health, substance dependence, personal finances, and criminal offending outcomes, following a gradient of self-control.” (p. 2693)	The authors first talk about self-control intervention programs to boost health and wealth and reduce crime. Given that intervention programs that increase self-control would be effective (“reap benefits”) only if self-control not only predicted but also caused these outcomes, the research question seems to be causal. The question about whether self-control is “important for the health, wealth, and public safety of the population” is vague. The subsequent sentence about prediction and the title of the article suggest that the study intends to investigate whether self-control is an important variable when one intends to predict health, wealth, and public safety.
Introduction	“Policy-making requires evidence that isolates self-control as the active ingredient affecting health, wealth, and crime, as opposed to other influences on children’s futures, such as their intelligence or social class origins. Dunedin study data allowed the requisite statistical controls for IQ and social class.” (p. 2694)	The terms “active ingredient” and “affecting” suggest that what is needed is causal knowledge. The term “causal” is absent. In addition, whereas they suggest that policymakers need causal knowledge, they do not explicitly state whether the goal of the study is to actually provide such knowledge.
Comments section (i.e., Discussion section)	“It was possible to disentangle the effects of children’s self-control from effects of variation in the children’s intelligence, social class, and home lives of their families, thereby singling out self-control as a clear target for intervention policy.” (p. 2697)	The statement that self-control is a clear target for intervention policy suggests that a “causal effect” is intended.
Example 2 (Lüdtke, Roberts, Trautwein, & Nagy, 2011)		
Current Research section	“Approximately 2,000 German students were tracked over 4 years from high school to university or to vocational training or work. . . . First, the experience of life events over the 4 years of the study should be predicted by standing on personality traits at Time 1 (T1). . . . Second, in line with the results reported by Vaidya et al. (2002), we hypothesized that experiencing more positive events would be associated with increases in extraversion, whereas experiencing negative events would be associated with increases in neuroticism.” (p. 622)	The research goals seem to be prediction (“should be predicted by standing on personality traits at Time 1”) and description (“we hypothesized that experiencing more positive events would be associated with increases in extraversion”).
Heading in Results section	“Life Paths and Personality Traits: Selection and Socialization” (p. 626)	In this section (and other parts of the article), the authors talk about “socialization effects”, which implies that the authors intend to investigate the causal effects of life paths (studying at a university vs. vocational track) and life events on the Big Five personality traits.
Discussion	“What was most compelling about our study of life events and their relation to personality development was how they provided insights going beyond any current theoretical ideas on why personality traits change in young adulthood.” (p. 631)	Only if the life events cause personality changes will they provide insights into *why* personality traits change. Hence, the interpretation of the findings in this passage is not in line with purely descriptive or predictive research goals.
Example 3 (Grosz et al., 2019)		
Current Research section	“First, mean-level changes in narcissistic admiration and Mach [Machiavellianism] during early adulthood were examined in both cohorts (TOSCA-2006 and TOSCA-2002). Second . . . we investigated associations between studying economics and changes in narcissistic admiration and Mach. Third . . . we investigated associations between life events and changes in narcissistic admiration and Mach.” (p. 470)	The first part of the study (mean-level changes) is descriptive. The second and third parts of the study also appear to be descriptive because the authors talk about investigating “associations.”
Method section	“We ran the studying economics and life events analyses with and without control variables. We included the control variables for two reasons. First, we included them to prevent spurious associations. For example, the initial level of self-esteem might be a confounder.” (p. 471)	The inclusion of control variables and the mentioning of “spurious associations” and “confounders” would make more sense if the goal were to estimate a causal effect than if the goal were to simply describe the associations.
Heading in Results section	“Experiences Related to Changes in Narcissistic Admiration and Machiavellianism During Early Adulthood (Socialization Effects)” (p. 475)	In this section (but also in other parts of the article), the authors talk about “socialization effects”, which implies that the authors intended to estimate the causal effects of the experiences (studying economics or a life event) on changes in narcissistic admiration and Machiavellianism.
Limitations section	“Finally, although we used the term socialization effect in this study in accordance with previous research on personality development, our data and analyses did not allow us to make causal claims.” (p. 480)	Here, the authors follow the standard practice in psychology to avoid drawing explicit causal inference on the basis of nonexperimental evidence and instead try to confine themselves to using descriptive language.
Example 4 (Cheng, Tracy, Foulsham, Kingstone, & Henrich, 2013, Study 1)		
Title	“Two Ways to the Top: Evidence That Dominance and Prestige Are Distinct Yet Viable Avenues to Social Rank and Influence” (p. 103)	The goal is to investigate whether dominance and prestige are avenues to social rank and influence (i.e., whether dominance and prestige have a causal effect on social rank).
Abstract	“In 2 studies, we investigated the impact of 2 fundamental strategies—Dominance (the use of force and intimidation to induce fear) and Prestige (the sharing of expertise or know-how to gain respect)—on the attainment of social rank.” (p. 103)	This passage suggests that the study investigated the causal effect (“impact”) of the two strategies on social rank.
Current Research section	“In Study 1 we examined whether Dominance and Prestige spontaneously emerge and coexist as viable rank-attainment strategies within the same social groups, by asking previously unacquainted individuals to complete a collaborative task and allowing social hierarchies to naturally emerge.” (p. 109)	The phrase “rank-attainment strategies” might imply that the study investigates whether dominance and prestige have a causal effect on rank attainment.
Limitations and Future Research section	“One limitation of the present research is our reliance on a correlational approach, which prevents us from directly addressing questions of causality—whether Dominance and Prestige are causal antecedents to social rank.” (p. 120)	Here, the authors follow the standard practice in nonexperimental psychology to avoid addressing causal research questions straightforwardly.

To be clear, we do not intend to criticize the authors or the quality of these four articles; our intention is instead to criticize the norms regarding causal inference that these authors and most other nonexperimental psychologists adhere to. These norms permeate many aspects of psychological science, from the education of psychological researchers (e.g., causal-effect estimation based on nonexperimental evidence plays only a minor role in the methods and statistics curricula in psychology) to the review process at scientific journals (e.g., author guidelines, editors, and reviewers asking for the removal of causal language).

## Reasons Behind the Taboo

Why do psychologists think that it is legitimate to make explicit causal inferences on the basis of experimental evidence but not on the basis of nonexperimental evidence? Imagine that we wish to study the effect of a new therapy on the recovery of depressed people. In a purely observational study, we may observe that, relative to nontreated people, clients improve when undergoing treatment. Yet it is likely that people who underwent treatment differed from nontreated people with respect to background factors that determine recovery (e.g., age, education, financial resources, social support). Thus, we cannot directly infer that the therapy worked—changes in recovery rates might have been caused by the treatment or by other factors. In an experimental study, the randomized assignment to the treatment (i.e., therapy) and control conditions is intended to eliminate the causal link between the background factors and choice of treatment. Hence, the background factors cannot serve as an alternative explanation of a higher rate of recovery in the treatment group if the randomization is successful (e.g., Pearl, 2009).

However, the problems of multicausality that render nonexperimental evidence weak and potentially nondiagnostic are to some extent present in experimental research with randomized groups as well. This is because many treatment or experimental manipulations will affect not only the independent variable they are intended to affect. Even an obvious physical manipulation such as stimulus presentation time can have many causal effects at different levels of aggregation (e.g., by inducing time pressure or stress, undermining self-efficacy, inducing distinct cognitive strategies), and identifying the relevant mechanism might be challenging (Bullock, Green, & Ha, 2010). Hence, causal inference always goes beyond what is observed, and it always rests on assumptions (e.g., Waldmann, Hagmayer, & Blaisdell, 2006). Some philosophers have even argued that it is a top-down rather than a bottom-up endeavor that involves a priori world knowledge (e.g., Kant, 1781/2002).^1^ Taken together, although experimental designs are the method of choice for blocking the effects of background factors, causal inferences are speculative inferences regardless of whether the study is conventionally classified as nonexperimental or experimental.

## Consequences of the Taboo Against Explicit Causal Inference

### Impairment of study design and data analysis

The ambiguity in the goals of nonexperimental studies (see Table 1) brings about a distinct lack of careful and explicit causal reasoning in study design and data analysis. Nonexperimental psychologists will usually have a coarse mental representation of the causal network in which their variables of interest are embedded. That is, they usually have some assumptions about the causes and consequences of the variables they are studying and about the causal mechanisms and mediating processes that lead from the independent variable(s) to the dependent variable(s). Yet these assumptions about the underlying causal network are hardly ever spelled out explicitly. For example, many nonexperimental psychologists do not explicitly justify why they include certain control variables, and hardly any of them use formalized frameworks developed to support causal reasoning such as the *potential-outcome framework* (e.g., Morgan & Winship, 2015; Rubin, 2005) or *directed acyclic graphs* (DAGs; e.g., Pearl, 2009). As a consequence of this unstructured approach, researchers may forget to assess and control important confounding variables, or they may erroneously control for mediators and collider variables, hence introducing bias (e.g., Elwert & Winship, 2014; Foster, 2010b; Rohrer, 2018). This state of affairs was bemoaned by Foster (2010b) after he had edited the journal *Developmental Psychology* for 5 years: “Currently, developmentalists conduct complex analyses that are not useful in pursuing either aim: The analyses are too complex to produce good description, and the complexity is not employed in a manner that facilitates causal inference” (p. 1760).

Furthermore, the causal assumptions encoded in structural equation models are often ignored or at least not discussed openly. For instance, by setting a coefficient to zero in a structural equation model, one is assuming that one variable does not have a causal effect on another variable. But structural equation models are frequently used in nonexperimental research without any explicit discussion or justification of such causal assumptions. This is problematic because the credibility of a structural equation model depends on the credibility of its causal assumptions (e.g., Bollen & Pearl, 2013).

### The taboo holds back cumulative research

A further consequence of the reluctance to explicitly talk about causality is that our understanding of the underlying causal mechanisms progresses at a slow pace, if at all. This issue has been highlighted in the field of personality research, which, because of the nature of its research subject, relies heavily on nonexperimental data:

During the past 50 years, personality psychology has made considerable progress concerning personality description, and prediction of and by personality. In contrast, explanation of personality development and personality effects has lagged far behind. In the coming decades, much more inspiration and transpiration are needed to change this unsatisfactory situation. (Asendorpf et al., 2016, p. 305)

We believe it is currently difficult for fields strongly characterized by nonexperimental research to accumulate causal knowledge because most previous studies have not explicitly stated the causal link they have identified or the assumptions under which this link should hold. These assumptions can often be reconstructed indirectly only on the basis of the analyses the authors chose to apply. For example, controlling for a third variable implies that it is understood as a confounder rather than as a mediator of the effect of interest. Still, the assumptions about the underlying causal network will often remain opaque, and thus, the conditions under which a coefficient can (or cannot) be interpreted as a causal effect remain unclear.

This opaqueness enables undesirable flexibility (e.g., Eisenberg, 1984; Smaldino, 2017), which discourages cumulative research. If researchers do not clearly specify the causal effect they think they have identified, a study’s findings are hardly falsifiable. Imagine, for example, that Researcher A publishes a nonexperimental study on subjective well-being and relationship satisfaction and concludes that a person’s low subjective well-being causes relationship dissatisfaction in a romantic partner. Researcher B might read the article and disagree with the conclusion because Researcher B thinks the health of the person confounds the relationship between subjective well-being and the partner’s relationship satisfaction. Researcher B might then write a comment and criticize Researcher A’s study for not assessing and controlling for health, or Researcher B could conduct a new study to investigate whether the relationship still holds when controlling for health. On the other hand, if Researcher A had not explicitly claimed that the effect of subjective well-being on the partner’s relationship satisfaction was causal, Researcher B would have had a hard time pinning down what exactly to say about Researcher A’s study: “The study did not correctly answer the question it did not explicitly try to answer” is not a compelling criticism. If confronted with criticism, Researcher A could retreat to the position that the finding was descriptive to begin with, even if this particular reading of the study is probably less interesting. Being unclear about the purpose of a study opens the door to such motte-and-bailey strategies in which researchers profit from the more interesting but difficult-to-defend causal interpretation of their effect (the bailey), but once challenged, they retreat to the almost trivial yet difficult to attack descriptive finding (the motte).

No single study can test all assumptions and rule out all potential alternative causal explanations. A variety of study designs, data sources, and methods are needed to attain confidence in estimates of causal effects (e.g., Briley, Livengood, & Derringer, 2018; Hernán, 2018b; Lawlor, Tilling, & Davey Smith, 2016). Such a cumulative endeavor needs to explicitly consider the assumptions that are involved. If not, research may simply go around in circles or end up in a futile back and forth when nobody notices that their discrepant conclusions hinge on certain assumptions about which one could argue in a more fertile manner.

### Disconnect between original findings and their subsequent interpretation

The taboo against explicit causal reasoning and language has furthermore led to a disconnect between the original nonexperimental findings and their subsequent interpretation. Even if authors refrain from making causal interpretations in their original study, subsequent theoretical articles, reviews, or Introduction/Discussion sections will refer to the very same findings in a way that makes sense only if they were meant to be read as causal effects. The citing authors likely have no intention to mislead readers—they might simply have not considered the design of the respective study in great detail.

For example, the neosocioanalytic theory has posited, on the empirical basis of longitudinal research that did not explicitly estimate causal effects, that investments in age-graded social roles drive (i.e., cause) personality trait change (e.g., entering the workforce after education leads to increases in conscientiousness; e.g., Roberts & Wood, 2006). Theories are usually causal in nature because cause-and-effect relationships permeate the way we think and make sense of the world (e.g., Kant, 1781/2002; Waldmann et al., 2006). Hence, if empirical researchers in a field do not tackle causal questions explicitly and instead try to constrain themselves to descriptive or predictive statements and research questions, then a disconnect between empirical findings and theory is almost inevitable.

A similar disconnect can arise when nonexperimental studies are cited to make certain arguments in literature reviews and Introduction sections. For example, two recent reviews argued that intervention studies on how to change personality traits are vital and needed because personality traits predict important life outcomes in the domains of education, work, relationships, health, and well-being (Bleidorn et al., 2019; Roberts et al., 2017). The implicit assumption must be that personality traits *cause* the life outcomes; otherwise, changing the personality traits through interventions will not change the respective outcomes. It is possible that personality is indeed the cause; however, most previous empirical studies on the topic did not explicitly investigate these causal effects.

Engaging in “stealth causal inference” from a distance (i.e., assuming causal relationships on the basis of descriptive or predictive findings reported elsewhere) may be convenient for nonexperimental fields because it means that authors do not have to defend explicit causal claims, yet everybody gets to enjoy explanatory accounts and the impression of a deep understanding of the subject matter. However, the disconnect between original findings and subsequent causal interpretations renders arguments and theories—even those that seem to be supported by an impressive number of empirical studies—speculative, which limits their usefulness for researchers and policymakers alike.

For researchers, speculative arguments and theories are not very helpful for designing causally informative studies: Although speculation might stimulate new research ideas, it does not provide reliable information about which variables to assess and control for. Furthermore, the speculative nature of theories means that derived hypotheses have a lower prior probability of being true than the hypotheses derived from less speculative theories (i.e., theories with firmly established relationships and laws such as natural selection in Darwin’s theory of evolution). Their lower prior probability in turn results in more nonsignificant findings and more false-positive findings (Diekmann, 2011; Fiedler, 2017; Ioannidis, 2005).^2^ Hence, theories without firmly established relationships and laws are not particularly useful, for example, for tackling the replication crisis in psychology (Fiedler, 2017; see also Muthukrishna & Henrich, 2019).

For policymakers, theories are useful if they contain firmly established causal relationships because only then can policymakers design interventions that successfully tackle pressing issues in the world. Although predictive findings might help to identify at-risk groups that might want to be targeted by interventions (e.g., adolescents with learning disabilities or self-control issues), predictive findings do not inform policymakers about how they can intervene. We can thus understand one reason for the lamented lack of interventions and policies targeting personality traits (e.g., Bleidorn et al., 2019)—unless we establish that personality traits are indeed meaningful causes, why would one want to target them?

## Recommendations for Integrating Causality in a More Productive Manner

### Steps of causal inference in nonexperimental studies

How can we do better? Nonexperimental researchers should openly admit when their goal is causal inference—and then ensure that their study pursues this goal in a rigorous and transparent manner. The following four steps of causal inference might help them do so.

In Step 1, researchers should articulate a clear causal question and state the precise definition of the causal effect of interest. Translating the causal question into a hypothetical experiment and counterfactual thinking can help researchers do so because the counterfactual question “What would happen to an individual if one changed the treatment?” lies at the heart of causal inference (e.g., Foster, 2010a; Hernán, 2018a; Morgan & Winship, 2015). That is, the causal effect of interest is the difference between the outcome if the individual had experienced the treatment and the outcome if the individual had not experienced the treatment. Thinking about how things are for an individual and how things would be different if the individual had not experienced the treatment can be formally expressed using the potential-outcomes framework (for an accessible introduction, see Foster, 2010a; see also, e.g., Holland, 1986; Rubin, 2005).

In Step 2, researchers might want to think carefully about how other variables relate to the putative causal variable (i.e., the treatment) and outcome variable to identify potential confounders, colliders,^3^ mediators, and instrumental variables (see Box 1). The assumptions about this underlying causal web can be expressed in a DAG (e.g., Pearl, 2009). A DAG connects variables with arrows representing causal relationships. Note that the DAG should contain all relevant variables, not only the ones that are available, observable, or measurable. The DAG helps researchers align the study design and data analysis to the actual aim of the study (for accessible introductions to DAGs, see, e.g., Foster, 2010a; Rohrer, 2018). As a side note, whereas counterfactual thinking and DAGs may be new tools for many psychologists, they are in line with Campbell’s tradition of identifying plausible threats to internal validity (i.e., causal inference) and then including study design features and statistical adjustments that can potentially rule out those specific threats (e.g., Campbell, 1988; Matthay & Glymour, 2020; West & Thoemmes, 2010).

Box 1. **What Is Instrumental-Variable Estimation?**

Instrumental-variable estimation is a method for estimating the causal effect of the treatment *X* on the outcome *Y* with the help of an instrumental variable *Z*. An instrumental variable *Z* is a variable that is associated with the treatment, and only because of its association with the treatment is it associated with the outcome. More specifically, an instrumental variable should fulfill the following four assumptions:

1. The *relevance* assumption: The instrument *Z* and treatment *X* are associated either because *Z* has a causal effect on *X* (left panel) or because *X* and *Z* share a common cause U* (right panel).
2. The *exclusion* restriction: *Z* affects the outcome *Y* only through *X*.
3. The *exchangeability* assumption (also called *independence* assumption): *Z* does not share common causes with *Y* (other than U*).
4. The *monotonicity* assumption: *Z* cannot increase *X* for some individuals and decrease it for others (e.g., Bollen, 2012; Labrecque & Swanson, 2018; Lousdal, 2018).

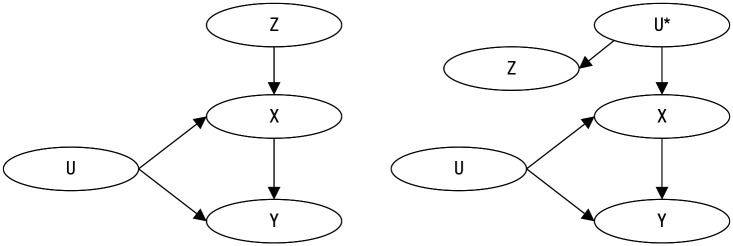

The assumptions can only partially be tested empirically and require theoretical justification (Labrecque & Swanson, 2018). If an instrument that meets these assumptions can be identified, the causal average effect of *X* on *Y* can be estimated even in the presence of unmeasured confounding *U*. A variable that does not fulfill the second and third assumptions can be transformed into a variable that fulfills these assumptions by adjusting for confounding variables.

For a continuous-treatment variable, the estimand for the instrumental variable is the ratio

$$\frac{\mathrm{Cov}(Y,\;Z)}{\mathrm{Cov}(X,\;Z)}.$$

Different types of instruments have been proposed: researcher-induced randomization (e.g., a randomized antismoking intervention is the instrumental variable Z and smoking is the treatment variable X), natural randomization processes (e.g., Mendelian randomization, in which alleles are allocated at random in offspring), and natural variation (e.g., preference for treatment according to the availability of a facility or physician; e.g., Bollen, 2012; Lousdal, 2018).

Step 3 involves establishing an identification strategy and estimating the causal effect. That is, given the assumptions from the previous steps, researchers derive a way to estimate the causal effect without bias from the data at hand. For example, this could involve a multiple regression model if all relevant confounding variables are available in the data, or it could involve the use of instrumental-variable estimation if un-observed confounding is assumed (for introductions to and discussions of various identification strategies, see Box 1; also see Foster, 2010a; MacKinnon & Pirlott, 2015; Mõttus & Kandler, 2018; Pingault et al., 2018; Rutter, 2007). Further inspiration for methods that can be used to investigate causal relationships on the basis of nonexperimental data can be found in fields such as economics, political science, or sociology. Parts of economics, political science, and sociology have embraced the challenge of causal inference on the basis of nonexperimental evidence, for example, through the use of instrumental-variable estimation (see Box 1), regression-discontinuity designs, or fixed-effects models (e.g., Allison, 2009; Angrist & Pischke, 2008; Gangl, 2010; Morgan & Winship, 2015). All of these approaches have their own pitfalls, but psychologists are lucky that they can learn from critical discussions that have already transpired in other fields of research. Once the identification strategy is in place, it can be used to estimate the causal effect.

In the last step, Step 4, researchers test their identification strategy against violations of assumptions to see how much the effect estimate would change if certain assumptions were violated. For example, if the assumption is that all confounders have been observed, a researcher might want to compute what would happen if unobservable variables were to confound the effect (for more information on sensitivity analysis, see, e.g., Frank, Maroulis, Duong, & Kelcey, 2013; Greenland, 1996; Rosenbaum, 2005; Rosenberg, Xu, & Frank, 2019; VanderWeele & Ding, 2017). The last step should also involve a discussion of potential alternative explanations for the observed effect. This discussion, along with future directions for research, might be provided in the Discussion section.

In Boxes 2 and 3 and Figures S1 and S2 in the Supplemental Material available online, we briefly illustrate these four steps of causal inference with research questions from the four articles presented in Table 1. Please note that a detailed description and exemplification of all steps is beyond the scope of the current article (for more details on steps of causal inference, see Foster, 2010a).

Box 2. **Brief Illustration of the Steps of Causal Inference for Example 1 (Moffitt et al., 2011)**

***Step 1 (basic definitions)***. Childhood self-control is the treatment variable that causes the outcome adulthood physical health. Self-control is defined as the ability to delay gratification, control impulses, and modulate emotional expression. Physical health is defined as cardiovascular, inflammatory, respiratory, dental, and sexual health (Moffitt et al., 2011).

***Step 2 (causal network)***. The directed acyclic graph (DAG) below illustrates the causal relationships we assumed on the basis of previous research. For example, serotonin levels in the central nervous system are believed to have a genetic basis, to be alterable by life circumstances, to affect conscientiousness (i.e., which is often seen as synonymous with self-control), and to help regulate the core bodily functions (appetite and sleep) that are necessary for good health (e.g., Carver, Johnson, Joormann, Kim, & Nam, 2011; Caspi, Hariri, Holmes, Uher, & Moffitt, 2010; Friedman et al., 2014). Genes, childhood socioeconomic status (SES1), and childhood serotonin are confounders because they have independent causal paths to youth self-control (SC1) and adulthood physical health. In the DAG, the numbers after the variables indicate the time period. SC = self-control; SES = socioeconomic status; Snares = harmful lifestyles—e.g., started smoking, unplanned pregnancy.

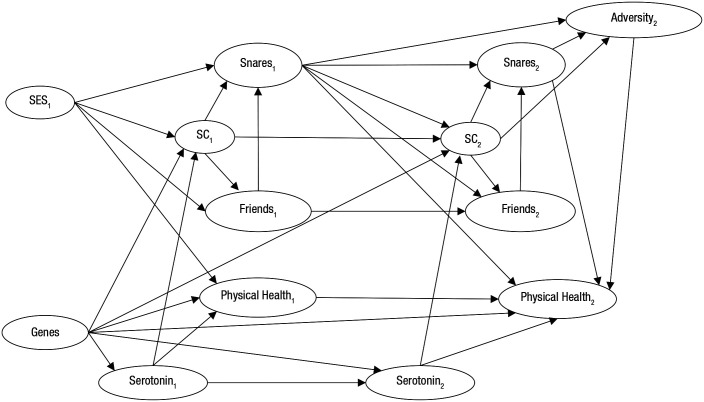

***Step 3 (identification strategy)***. An approach that could be applied to block the confounding paths involving genes, SES1, and childhood serotonin and thus achieve an unbiased estimate might be to run a study with monozygotic twins who are raised in the same family (i.e., pairs of individuals with identical genes and socioeconomic background). We would estimate the causal effect by regressing the intratwin difference in adulthood health on the intratwin difference in childhood self-control and the intratwin difference in childhood serotonin. Differencing blocks the paths via the node genes and SES1 under the assumption that genes and SES1 influence both individuals of a twin pair in the same way (e.g., Allison, 2009; Campbell & Kenny, 1999; Kim & Steiner, 2019). Controlling for the intratwin difference in childhood serotonin would neutralize the confounding effect of childhood serotonin.

***Step 4 (probing assumptions and alternative explanations)***. The validity of the identification strategy depends on whether all confounding twin-varying variables were included in the DAG and properly adjusted for in the analysis. For example, the intratwin differences in childhood self-control might have been caused by intratwin differences in adverse childhood experiences that also caused intratwin differences in childhood intelligence, and childhood intelligence (not childhood self-control) might have been the actual cause of adulthood health. Thus, as a robustness check, we would add the intratwin difference in childhood intelligence as a control variable in the regression.

Box 3. **Brief Illustration of the Steps of Causal Inference for Example 2 (Lüdtke, Roberts, Trautwein, & Nagy, 2011)**

***Step 1 (basic definitions)***. Studying at a university (as opposed to vocational training or work) at around the age of 20 to 25 is the treatment variable that causes the outcome conscientiousness at around the age of 25. Conscientiousness is defined as a personality trait characterized by the propensity to follow socially prescribed norms for impulse control, to be goal-directed, to plan, and to be able to delay gratification (Roberts, Jackson, Fayard, Edmonds, & Meints, 2009).

***Step 2 (causal network)***. The directed acyclic graph below illustrates the causal relationships we assumed on the basis of previous research. For example, Spiess and Wrohlich (2010) suggest that the distance to the nearest university affects the probability of enrolling in higher education. In the DAG, the numbers after the variables indicate the time period. Consc. = conscientiousness; GPA = high school grade point average; IQ = intelligence; SES = socioeconomic status; Snares = harmful lifestyles—e.g., started smoking, unplanned pregnancy.

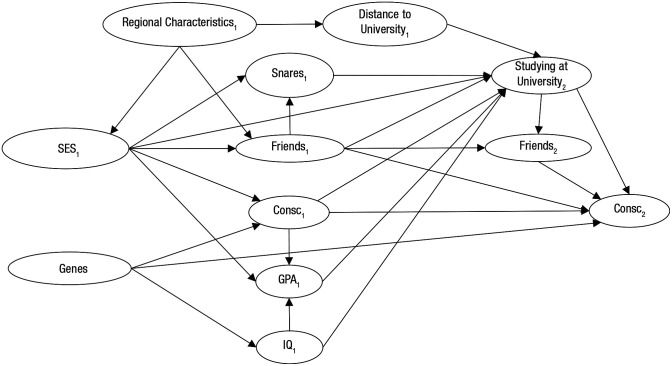

***Step 3 (identification strategy)***. We propose that distance to the nearest university be used as an instrumental variable (for details, see Box 1). That is, we would utilize the fact that distance to the nearest university causally affects studying at a university while there are hardly any other paths from distance to the nearest university to conscientiousness. The alternative paths all go through regional characteristics of the place of origin: Places far from any university might be poorer or more rural. The poorness or ruralness of a place might affect both whether there is a university and the likelihood of studying via the socioeconomic status of the family of the participant (e.g., parents’ education). Thus, we would control for the regional characteristics (indicators of urbaneness and prosperity of the place of origin) in the instrumental-variable estimation.

***Step 4 (probing assumptions and alternative explanations)***. One assumption is that all paths from distance to the nearest university to adulthood conscientiousness go through studying at a university and regional characteristics. To probe this assumption, we would regress youth conscientiousness on distance to the nearest university and regional characteristics. If distance to the nearest university were incrementally associated with youth conscientiousness, this would suggest that there are paths from distance to the nearest university to adulthood conscientiousness that are not mediated by regional characteristics or studying at a university, which would bring into question the validity of the instrumental variable (i.e., distance to the nearest university).

### Further recommendations

Whereas the details of every particular attempt of causal inference will necessarily vary, we advise psychologists to be explicit about the entire process. Researchers should state that they are trying to estimate a causal effect, and they should be clear about the assumptions underlying their analyses. Being open about causality invites more critical reflection about the underlying assumptions, which may also open the door for more refined and productive rebuttals as points of disagreement can be pinpointed. To cite Charles Darwin (1981/1871):

False facts are highly injurious to the progress of science, for they often endure long; but false views, if supported by some evidence, do little harm, for everyone takes a salutary pleasure in proving their falseness; and when this is done, one path towards error is closed and the road to truth is often at the same time opened. (p. 385)

Likewise, we advise researchers to make explicit rather than implicit causal-inference statements in the arguments and theories they present in their Introduction and Discussion sections, reviews, and theoretical articles. This does not mean that they should make bold causal claims when there is substantial uncertainty. Instead, they should simply be more transparent about when an argument or theory depends on the existence of a particular causal effect (rather than just a correlation), and they should discuss the extent to which previous studies have provided compelling evidence for it. To do so, it might be helpful to state whether a causal effect in a theory or argument rests on previous experimental or nonexperimental evidence.

Finally, we suggest that the field as a whole should try to shift its norms toward a more productive engagement with causal inference on the basis of nonexperimental data. Statistics and methods teachers could dedicate some more time to the topic—it may be time well spent because a clearer framework for causal inference makes it easier to talk about a broad range of topics, such as missing data problems (Thoemmes & Mohan, 2015) and threats to validity, which affect most types of research (Matthay & Glymour, 2020). Editors and reviewers may also encourage a shift in thinking. By no means should they let their guard down and allow researchers to confuse correlation with causation. However, instead of simply policing language or requesting boilerplate statements about limitations, they might ask hard questions—about the actual goal of the study (e.g., asking for clarification about why mere prediction would be interesting or highlighting discrepancies between supposedly noncausal questions and the discussed implications), about the authors’ understanding of the underlying causal web (e.g., requesting that the authors provide a DAG to justify their choice of covariates), or about more specific recommendations for future studies (e.g., if an experimental clarification is suggested, there should be some discussion about what a feasible experiment could look like). In some cases, authors may actually feel confident enough to make a causal claim—if it is accompanied by a transparent discussion of the underlying assumptions, then readers are given the information they need to form their own opinions.

## Conclusion

Causal inference on the basis of observational data is very difficult. However, this is not a good reason to render explicit causal inference taboo. Similar to when sex or drugs are made taboo, making explicit causal inference taboo does not stop people from doing it; they just do it in a less transparent, regulated, sophisticated, and informed way. Thus, we think it is about time that psychologists begin to talk openly about causality in nonexperimental research.

## Supplemental Material

Grosz_Supplemental_Material – Supplemental material for The Taboo Against Explicit Causal Inference in Nonexperimental Psychology Click here for additional data file. Supplemental material, Grosz_Supplemental_Material for The Taboo Against Explicit Causal Inference in Nonexperimental Psychology by Michael P. Grosz, Julia M. Rohrer and Felix Thoemmes in Perspectives on Psychological Science

## Transparency

*Action Editor:* Laura A. King

*Editor:* Laura A. King

## Notes

1. We thank Klaus Fiedler for bringing up this point.

2. If the prior probability for each of 10,000 hypotheses H1 is .04, then there are 320 true positives and 480 false positives, assuming a statistical power of .80 and an α level of .05. If the prior probability is .20, then there are 1,600 true positives and 400 false positives. If the prior probability is .40, then there are 3,200 true positives and 300 false positives (Diekmann, 2011; Fiedler, 2017).

3. A collider is a variable that is causally influenced (either directly or indirectly) by the treatment and the outcome. Adjusting for a collider can introduce a spurious (i.e., noncausal) association between the treatment and the outcome (e.g., Elwert & Winship, 2014; Rohrer, 2018).
