# Supplementary material for: The Taboo Against Explicit Causal Inference in Nonexperimental Psychology
Source: Perspect Psychol Sci. 2020 Jul 29;15(5):1243–55. doi: 10.1177/1745691620921521 (PMC7472833; doi:10.1177/1745691620921521)
Supplement: Grosz_Supplemental_Material – Supplemental material for The Taboo Against Explicit Causal Inference in Nonexperimental Psychology [file Grosz_Supplemental_Material.pdf]

# Supplemental Material

*Step 1 (Basic definitions)*. Studying economics at a university is the treatment variable that causes Machiavellianism (Mach) in later life. Studying economics is defined as studying business, macroeconomics, or another economics major as opposed to choosing a noneconomics major (e.g., engineering, sociology). Mach is defined as a personality trait characterized by cynical world view, pragmatic morality, cold rationality, deviousness, and a belief in the effectiveness of (one’s own) manipulative tactics (e.g., Christie & Geis, 1970; Jones & Paulhus, 2009).

*Step 2 (Causal network)*. The DAG below illustrates the causal relationships we assumed mainly on the basis of previous research.

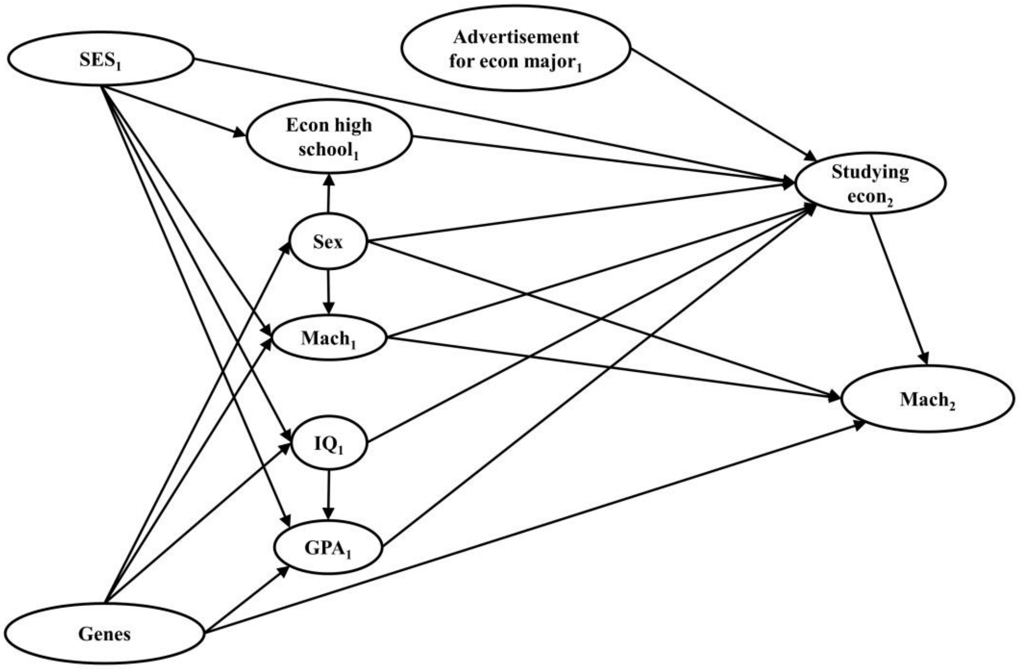


*Note*. The numbers behind the variables indicate the time period. SES = socioeconomic status; GPA = grade point average.

*Step 3 (Identification strategy)*. To attain a suitable instrumental variable for estimating the causal effect of studying economics on Mach, we would randomly assign participants to one of two conditions. In the experimental condition, participants would receive an advertisement about studying economics at a university in their final year of high school. In the control condition, participants would receive an advertisement about studying noneconomic majors. The random assignment to condition ensures that the advertisement is not confounded with other causes of Mach (see DAG). Furthermore, we assume that the advertisement affects the level of Mach after studying at a university only via studying economics. Admittedly, the advertisement might also have an effect on the level of Mach immediately after the advertisement, but this effect should be short-lived (i.e., temporary state changes in Mach) and should thus not influence the level of Mach after studying at a university.

*Step 4 (Probing assumptions and alternative explanations).* A potential limitation of the design is that those who are swayed by the advertisement might also be those who are swayed by the study major to change their personality (i.e., either due to the content of the courses or due to influences from peers). Thus, the estimation of the causal effect might only be valid for the subpopulations that responded to the advertisement (i.e., local average treatment effect) but not for other subpopulations of young adults. Another potential limitation might be that the advertisement has a very weak effect on choice of study major ("weak instrument"), which could lead to biased and inconsistent estimates (e.g., Bound, Jaeger, & Baker, 1995; see also the relevance assumption in Figure 1). Furthermore, if the advertisement backfires among some participants (e.g., if the provided information makes studying economics less attractive for some participants), the monotonicity assumption underlying the instrumental variable is violated (Figure 1).

***Box S1.*** Brief illustration of the steps of causal inference for Example 3 (Grosz et al., 2019).

| |
|---|
| *Step 1 (Basic definitions)*. Dominance is the treatment variable that causes the outcome social rank. Dominance is defined as the use of force and intimidation to induce fear in others (Cheng et al., 2013). Social rank is defined as the amount of social influence on others and attention from others in face-to-face groups. |
| *Step 2 (Causal network)*. The DAG below illustrates the causal relationships we assumed mainly on the basis of previous research. For example, previous research suggests that the height and muscularity of an individual might cause both dominance and social rank in face-to-face groups (e.g., Melamed, 1992; Blaker & Van Vugt, 2014). 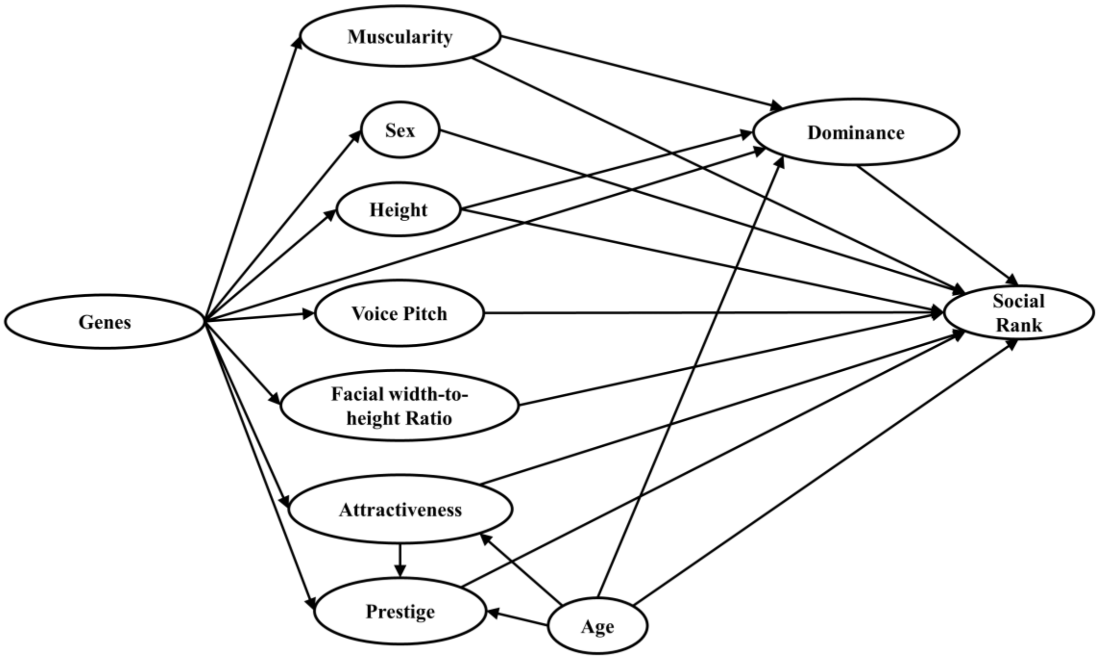  |
| *Step 3 (Identification strategy)*. We propose that the paths that might confound the estimation of the causal effect be blocked. We would block the path from sex to social rank by using only same-sex groups as Cheng et al. (2013) did. We would block the other paths by including muscularity, height, voice pitch, facial width-to-height ratio, physical attractiveness, prestige, and age as control variables in a multilevel model—we would use multilevel modeling because each individual is nested in a face-to-face group (Cheng et al.). |
| *Step 4 (Probing assumptions and alternative explanations)*. Because the identification strategy is to condition on observable variables, it is particularly important that all relevant variables are in the DAG. If a relevant variable is not part of the DAG, this might invalidate the identification strategy. For example, if physical health would have a causal effect on both dominance and social rank, then physical health might confound the association between dominance and social rank. Another assumption is that all variables in the model were assessed without measurement error. This assumption might not be valid for some variables (e.g., dominance, social rank, muscularity). We would check the robustness of our estimation by running the multilevel analysis in a structural equation model and correcting for measurement error by using latent variable modeling (e.g., Westfall & Yarkoni, 2016). |

***Box S2.*** Brief illustration of the steps of causal inference for Example 4 (Cheng et al., 2013).

## References


Blaker, N. M., & van Vugt, M. (2014). The status-size hypothesis: How cues of physical size and social status influence each other. In J. T. Cheng, J. L. Tracy, & C. Anderson (Eds.), *The psychology of social status* (pp. 119–137). New York, NY: Springer Science + Business Media. https://doi.org/10.1007/978-1-4939-0867-7_6

Bound, J., Jaeger, D. A., & Baker, R. M. (1995). Problems with instrumental variables estimation when the correlation between the instruments and the endogenous explanatory variable is weak. *Journal of the American Statistical Association, 90,* 443–450. https://doi.org/10.1080/01621459.1995.10476536

Cheng, J. T., Tracy, J. L., Foulsham, T., Kingstone, A., & Henrich, J. (2013). Two ways to the top: Evidence that dominance and prestige are distinct yet viable avenues to social rank and influence. *Journal of Personality and Social Psychology, 104,* 103–125. https://doi.org/10.1037/a0030398

Christie, R., & Geis, F. (1970). *Studies in Machiavellianism*. New York, NY: Academic Press.

Jones, D. N., & Paulhus, D. L. (2009). Machiavellianism. In M. R. Leary & R. H. Hoyle (Eds.), *Individual differences in social behavior* (pp. 93–108). New York, NY: Guilford Press.

Melamed, T. (1992). Personality correlates of physical height. *Personality and Individual Differences, 13,* 1349–1350. https://doi.org/10.1016/0191-8869(92)90179-s

Westfall, J., & Yarkoni, T. (2016). Statistically controlling for confounding constructs is harder than you think. *PLOS ONE, 11*(3), Article e0152719. https://doi.org/10.1371/journal.pone.0152719
